# Supplementary material for: Flexible Symmetric-Defection Antenna with Bending and Thermal Insensitivity for Miniaturized UAV
Source: Micromachines (Basel). 2024 Jan 21;15(1):159. doi: 10.3390/mi15010159 (PMC10818624; doi:10.3390/mi15010159)
Supplement: Supplementary file 1 [file micromachines-15-00159-s001.zip › micromachines-2818874-supplementary.pdf]

Supplementary Materials

Flexible Symmetric-Defection Antenna with Bending and Thermal Insensitivity for Miniaturized UAV

Xueli Nan [1,2], Tongtong Kang [1], Zhonghe Zhang [3], Xin Wang [1], Jiale Zhang [1], Yusheng Lei [4], Libo Gao [4], Jianli Cui [5,*] and Hongcheng Xu [6,*]

[1] School of Automation and Software Engineering, Shanxi University, Taiyuan 030006, China; nanxueli@sxu.edu.cn (X.N.); 202123603013@email.sxu.edu.cn (T.K.); 202123601010@email.sxu.edu.cn (X.W.); 202123603033@email.sxu.edu.cn (J.Z.)

[2] School of Biomedical Engineering, Shanghai Jiao Tong University, Shanghai 200030, China

[3] College of Electronics and Information Engineering, Shenzhen University, Shenzhen 518061, China; zzh422384683@163.com

[4] Pen-Tung Sah Institute of Micro-Nano Science and Technology, Xiamen University, Xiamen 361102, China; 19920231151619@stu.xmu.edu.cn (Y.L.); lbgao@xmu.edu.cn (L.G.)

[5] School of Physics and Electronics Engineering, Yuncheng University, Yuncheng 044000, China

[6] School of Instrument Science and Technology, Xi'an Jiaotong University, Xi'an 710049, China

* Correspondence: cuijianli@ycu.edu.cn (J.C.); hongc187@outlook.com (H.X.); Tel.: +86-183-347-89099 (J.C.); +86-187-341-26627 (H.X.)

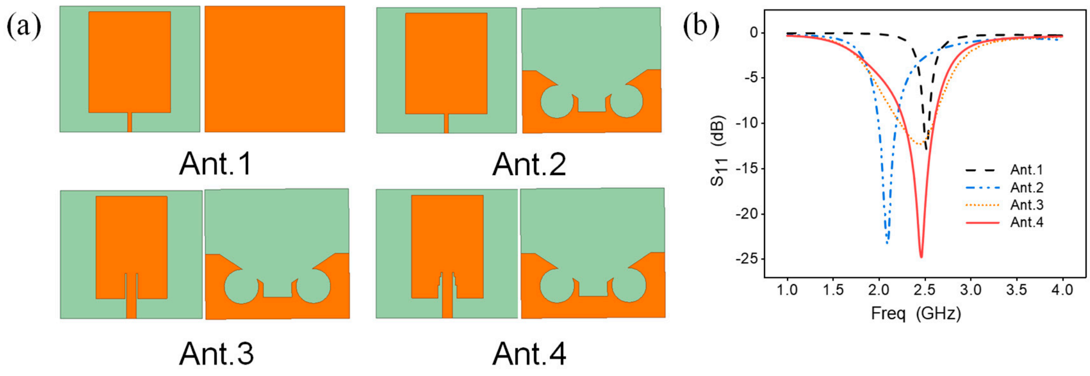

Figure S1 (a) Four antennas with different structure designs. (b) Return loss results of four antennas.

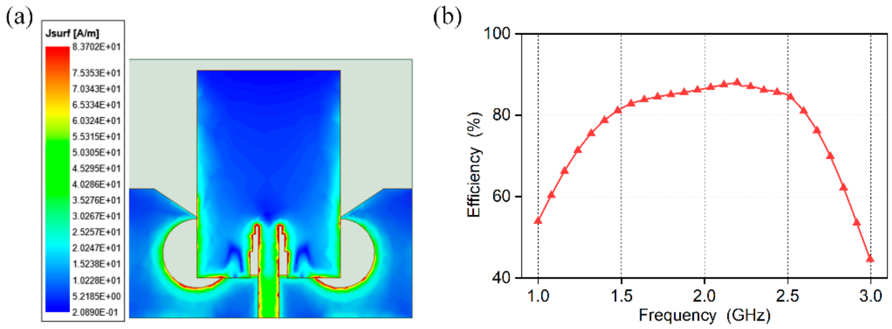

Figure S2 (a) Surface current distribution of FSDA at 2.45 GHz. (b) Radiation efficiency.

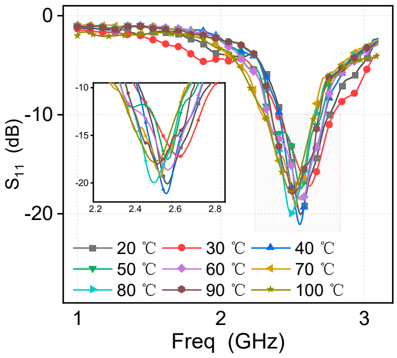

Figure S3 Return loss changes versus the temperature.

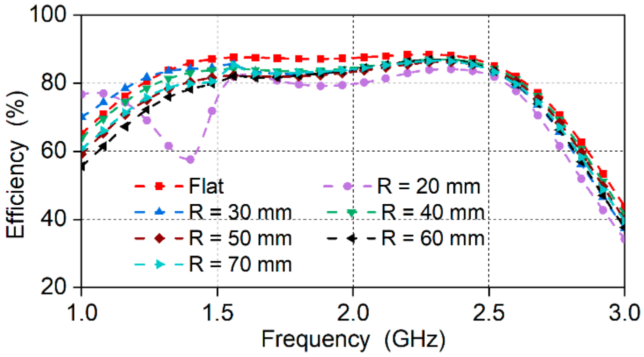

Figure S4 Radiation efficiency at various bending radii and flat.

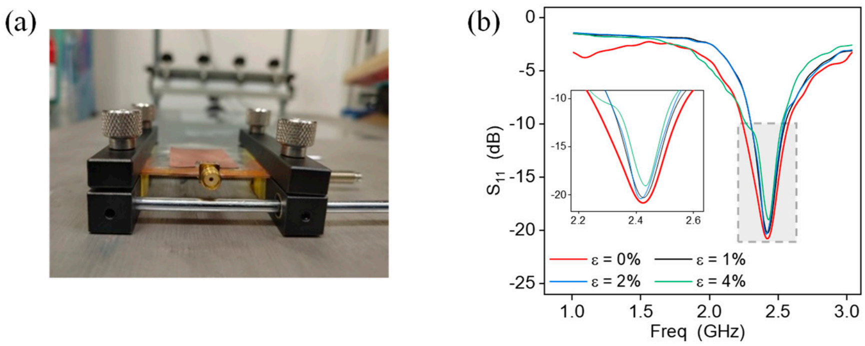

Figure S5 FSDA's stretching performance. (a) Stretching control machine. (b) Return loss changes of the FSDA versus different stretching strains.

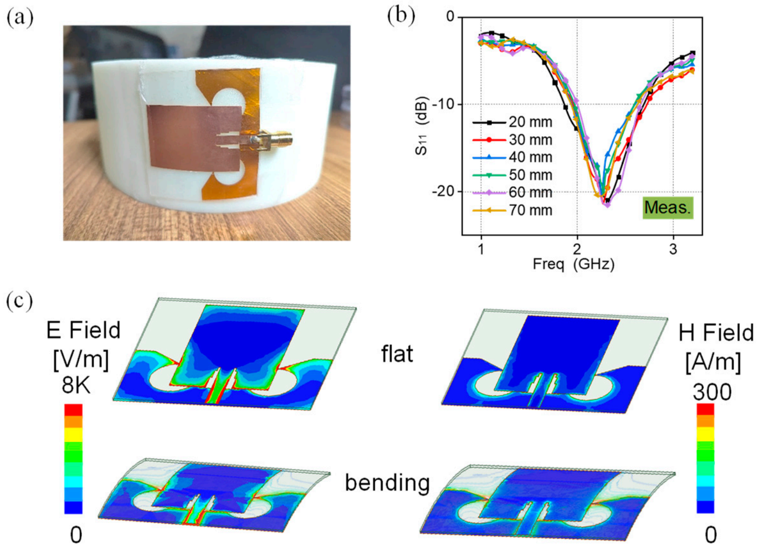

Figure S6 FSDA's bending performance (E-plane). (a) Optical image when the FSDA is attached to a cylinder surface along with the circumference direction. (b) Measured results for S_{11} with different bending radii. (c) Electric and magnetic field comparison while the FSDA is flat (top) and bending (bottom).

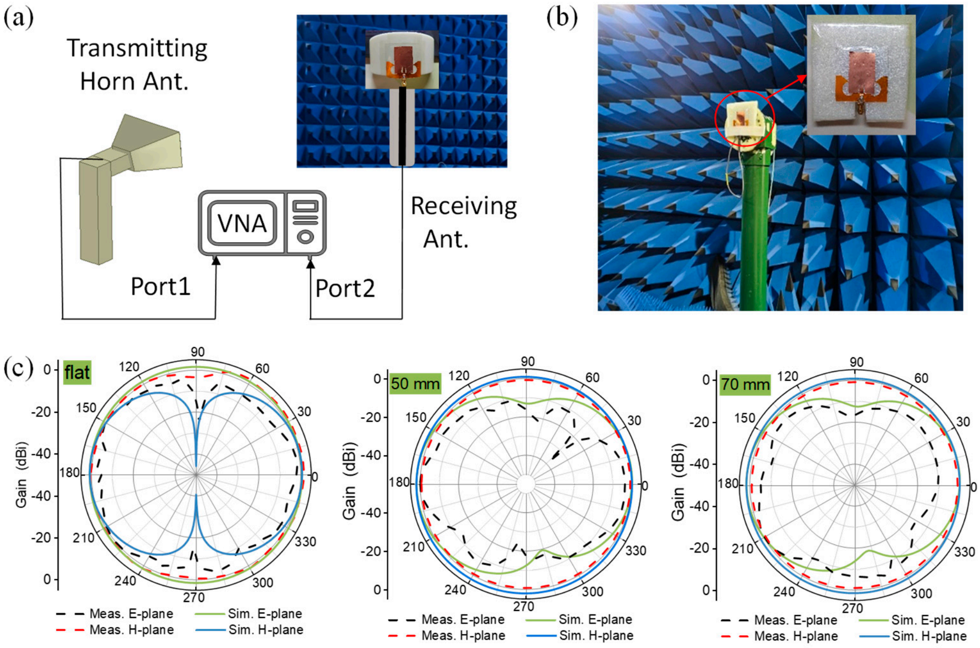

Figure S7 Comparison of the radiation patterns. (a) Schematic illustration of the free space testing. (b) Optical image of the tested antenna at a rotating platform in the anechoic chamber. (c) Measured and simulated radiation patterns at the H-plane and E-plane of the FSDA under three states, flat (left), conformal cylinder with a radius of 50 mm (middle), and 70 mm (right).

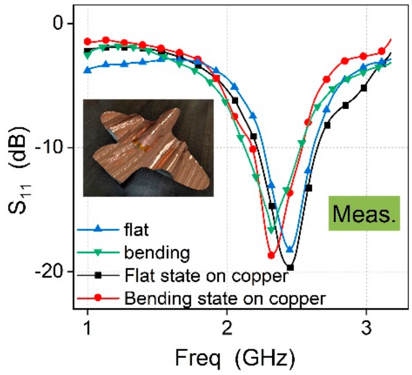

Figure S8 Measured results for the return loss while the antenna is tested with different statues and an optical image of the antenna mounted on the copper-covered 3D-printed UAV.
